# Pain when walking: individual sensory profiles in the foot soles of torture victims - a controlled study using quantitative sensory testing

**DOI:** 10.1186/1472-698X-12-40

**Published:** 2012-12-31

**Authors:** Karen Prip, Ann L Persson, Bengt H Sjölund

**Affiliations:** 1 Rehabilitation and Research Centre for Torture Victims, Copenhagen, Denmark; 2 Institute of Public Health, University of Southern Denmark, Odense, Denmark; 3 Beritta Gurrisgatan 21, Malmö, SE, 21775, Sweden

**Keywords:** Chronic pain, Falanga, Nerve injury, Sensitization, Torture

## Abstract

**Background:**

With quantitative sensory testing (QST) we recently found no differences in sensory function of the foot soles between groups of torture victims with or without exposure to falanga (beatings under the feet). Compared to matched controls the torture victims had hyperalgesia to deep mechano-nociceptive stimuli and hypoesthesia to non-noxious cutaneous stimuli. The purpose of the present paper was to extend the group analysis into individual sensory profiles of victims’ feet to explore possible relations between external violence (torture), reported pain, sensory symptoms and QST data to help clarify the underlying mechanisms.

**Methods:**

We employed interviews and assessments of the pain and sensory symptoms and QST by investigators blinded to whether the patients, 32 male torture victims from the Middle East, had (n=15), or had not (n=17) been exposed to falanga. Pain intensity, area and stimulus dependence were used to characterize the pain. QST included thresholds for touch, cold, warmth, cold-pain, heat-pain, deep pressure pain and wind-up to cutaneous noxious stimuli. An ethnically matched control group was available.The normality criterion, from our control group data, was set as the mean +/− 1.28SD, thus including 80% of all values.QST data were transformed into three categories in relation to our normality range; hypoesthesia, normoesthesia or hyperesthesia/hyperalgesia**.**

**Results:**

Most patients, irrespective of having been exposed to falanga or not, reported severe pain when walking. This was often associated with hyperalgesia to deep mechanical pressure. Hypoesthesia to mechanical stimuli co-occurred with numbness, burning and with deep mechanical hyperalgesia more often than not, but otherwise, a hypoesthesia to cutaneous sensory modalities did not co-occur systematically to falanga, pain or sensory symptoms.

**Conclusion:**

In torture victims, there seem to be overriding mechanisms, manifested by hyperalgesia to pressure pain, which is usually considered a sign of centralization. In addition there was cutaneous hypoesthesia, but since there was no obvious correlation to the localization of trauma, these findings may indicate centrally evoked disturbances in sensory transmission, that is, central inhibition. We interpret these findings as a sign of changes in central sensory processing as the unifying pathological mechanism of chronic pain in these persons.

## Background

Many victims of torture, even after extended time periods, experience pain in the feet when walking. The use of falanga (beatings on the soles of the feet) is a torture method which deliberately aims at inflicting intense pain in the feet and lower legs [1,2]. It is nowadays used systematically as a torture method especially in the Middle East and the Far East [3]. The foot pain often persists years after torture [2,4,5], and contributes to severe disabilities [6], even when walking moderate distances.

In previous studies we have confirmed and extended the clinical evidence of chronic foot pain and disability in falanga victims with chronic pain in the feet [5,6]. Chronic pain is also common after generalized torture. Olsen at al. [7] found that more than 80% of patients referred for rehabilitation of torture sequaele reported chronic pain. Similar figures have been reported for war veterans with post-traumatic stress disorders (PTSD; [8]). In addition, Defrin et al. [9] recently found that persons with PTSD may exhibit altered sensory processing.

When examining torture victims with quantitative sensory testing (QST), we recently and remarkably found no obvious differences in sensory function of the foot soles between groups of torture victims with or without exposure to falanga [10]. This was so in spite of the fact that almost all the falanga victims had moderate to strong pain in their feet and in twice as large an area of their foot soles as those torture victims who had not been exposed to falanga. One third of the latter did not report pain in their feet at all and many reported only slight pain. On the other hand, when compared to matched controls, torture victims had hyperalgesia to deep mechano-nociceptive stimuli, irrespective of exposure to falanga or to other forms of torture. Furthermore, cutaneous sensory fibre groups except those transmitting cold and heat pain seemed to be less sensitive to external stimuli. These findings are compatible with central changes of sensory transmission [11]. Thus, there seems to be a blend of chronic pain conditions in the feet of torture victims so that analyses of means at the group level do not reveal significant differences; thus the data should be analysed individually.

The primary objective of the present paper was to extend the group analysis of the QST data from our previous study [10] into individual sensory profiles of victims’ feet to explore possible relations between external violence (torture), reported pain, sensory symptoms and QST data to help clarify the underlying pain mechanisms [5,12].

## Methods

### Participants

The patients recruited were torture victims who had been granted asylum in Denmark. They were all referred to our centre from their general practitioner, because of their long-term sequelae from various types of torture that they had been subjected to several years earlier in their homeland. The patients were screened by an assessment team (physician, psychologist, physiotherapist and social worker), supported by an interpreter, with reference to the centre’s admission criteria: 1) torture victim with asylum in Denmark; 2) physical, psychological and social needs; 3) no overt psychosis; 4) no drug or alcohol abuse; and 5) available treatment capacity.

### Study selection criteria

The inclusion criteria for this study were tortured male patients originating from the Middle East and speaking Arabic or Farsi (the majority of currently referred male patients during the period May 2009 – June 2010). Seventy-nine consecutively referred patients were identified via the electronic patient records (see Flowchart, in [10]). One senior physician (BHS) screened these medical records according to the exclusion criteria: pathological structural changes in the feet and lower legs from reasons other than falanga, for example, sustained fractures, amputations, extensive scar tissue after burning or cuttings or foreign objects embedded in the feet such as shrapnel or bullets; nerve lesions in the lower legs from causes other than falanga, for example, diabetic or alcoholic polyneuropathy and also injury to the central nervous system such as stroke or spinal fractures. Twenty-seven persons were excluded, usually due to injury to the nervous system other than from falanga.

The reasons for exclusions were: rhizopathy (n=7), diabetes (n=3), not mentally fit (n=2), opioid medication (n=2), spinal fracture (n=1), arteriosclerosis in the legs (n=1), hydrocephalus (n=1), not been tortured but referred for having been secondarily traumatized (n=3), other problems and were referred for treatment elsewhere (n=7). Clinical symptoms and signs, including the typical temporal and anatomical progression of sensory symptoms of peripheral neuropathy were always sought for during the clinical history–taking and examination.

The remaining 52 patients were invited to participate in the study. At individual meetings the examiner (KP) informed them about the purpose and methods used in the study; seventeen persons turned down the offer at this stage. If the patient agreed to participate an informed consent form was signed and dates for the assessments arranged. Three persons started but dropped out during the test sessions. Thus 32 patients participated in all three sessions. All were offered compensation for travel expenses. Arabic or Farsi interpreters assisted at all sessions. The project was carefully introduced to the three interpreters involved, all of whom had long experience in interpreting for torture victims.

It was possible to recruit 14 ethnically and age matched men from the Middle East community in Copenhagen to form a control group, going through exactly the same QST methods as for the patients. They had a mean age of 37 years (range 21–55), had no chronic pain conditions, and were integrated and active in the Danish society. They had lived in Denmark for an average of 15.8 years (range 7–26) and all spoke Danish.

### Design

The examiner (KP) and a research assistant were blinded with regard to patient history, diagnosis and whether falanga torture had occurred or not. Breaking of the blinding took place only after the completion of all examinations and data analyses of the individual patients.

### Procedure and measurements

All examinations were scheduled to 3x2 hour sessions within a two-week period and took place in a quiet room (stable temperature of 22–24°C) in the research department at our centre. For details of the procedures and measurement techniques, we refer the reader to our previous paper [10].

### Test sites

To our knowledge only few studies have examined the arch of the foot sole with respect to normal QST values, and the available data are mostly found from the dorsum of the foot [13,14]. According to current standards for QST, measurements in neuropathic pain conditions should be performed in the area with the maximum pain [15] and we therefore chose the arch of the footsole under the intermediate cuneiform bone bilaterally for all sensory tests. In addition, this site has been reported to be the most sensitive in the foot sole [16].

### Assessments

#### Interview

The patients were asked about pain in the foot soles at rest and when walking and the findings were registered on a 3-point Likert scale (0=no pain, 1=slight/moderate pain, and 2=severe pain; cf. [5]) to determine activity related changes in foot pain. From these data the victims’ feet could be divided into three groups: no foot pain; stimulus-independent foot pain (pain appearing spontaneously at rest); and stimulus-evoked foot pain (pain evoked by activity, such as walking [5]). Reported sensory disturbances such as numbness, cold/burning, pricking or buzzing sensation were also registered.

#### Pain drawings

The patients shaded in the locations of their pain on a special foot chart (views of right and left foot soles; [17]). The shaded-in areas on the pain drawings were measured in square millimetres and calculated in percent of the total area using a commercial software programme (Quantify One; K:L:O:N:K, Denmark; [18]).

#### Pain intensity

Self-reported current pain intensity was assessed on a visual analogue scale (VAS; 0–100 mm; no pain=0; worst imaginable pain=100; [19]), with reference to each foot sole separately.

#### Quantitative sensory testing in the feet

For a detailed description of the techniques used to measure the different modalities, see our previous study [10]. Briefly, we measured mechanical detection thresholds (MDT) utilizing Semmes Weinstein monofilaments; cold detection thresholds (CDT) and warm detection thresholds (WDT), and also cold pain thresholds (CPT) and heat pain thresholds (HPT) utilising a thermal stimulator (MEDOC Inc., Israel); and deep mechano-nociceptive thresholds (pressure pain thresholds: PTT) using an electronic pressure algometer (Somedic, Höör, Sweden). In addition, temporal summation of mechano-nociceptive stimuli (wind-up pain) was examined by using the thickest Semmes-Weinstein monofilament (size 6.65; [20]). At 0.3 Hz the examiner applied the filament four consecutive times to the surface of the skin in the arch of each foot sole. The patients were asked to rate the pain intensity on a VAS after the 1^st^ and 4^th^ stimulus. A 5-minute pause followed. Thereafter, to produce a more intense stimulation, 10 consecutive stimuli were applied at the rate of 1.0 Hz. The patient rated their pain after the 1^st^ and 10^th^ stimulus. If the VAS difference between the 1^st^ and last stimulus was positive, a temporal summation (wind-up) had occurred.

Dynamic mechanical allodynia is a painful or unpleasant sensation evoked by a cutaneous mechanical stimulus which does not normally evoke pain. To examine this phenomenon we used light strokes with a soft brush (SENSELab™– Brush-05; Somedic, Hörby, Sweden). Three consecutive strokes over a 60 mm long distance in the arch of both foot soles were applied with the brush [21], and the patient indicated if the stimulus was unpleasant or painful.

Some of the QST stimuli that may evoke a transient unpleasant sensation or pain were applied as a pre-test in another area far from the feet (the right and left forearm [PPT], or in the palm of the hand [Windup-test]) to familiarize the patients with the procedures. It was emphasized that the QST test was not an endurance test and should not evoke pain; the patients should press the stop button or indicate to the examiner at the absolute first instance of pain detection. In this way the patient was in control at all times.

### Statistics

We chose to analyse the data from the right and left feet separately, since they may share common analysis mechanisms in the central nervous system, even if separate. The mean, SD, 95%CI, median, and range were calculated for all eight QST variables. The data were analyzed using the Statistical Package for Social Sciences (SPSS) version 18, Software for Windows (SPSS, Chicago; IL; USA).

### Ethics

Each participant was informed verbally about the study and was also given an information kit containing a comprehensive written description translated into their respective languages. They also received translated guidelines concerning participation in medical research issued by the Danish Ethical Committee. The assessments comply with the Helsinki II Declaration [22] and the patients could withdraw from the study at any time, without any impact on their planned rehabilitation at our clinic. The study was approved by the Research Ethics Committee in Region Copenhagen, Denmark (H-D-2009-068) and registered in the Danish Data Protection Agency.

## Results

### General

All 32 patients reported pain in many parts of the body. When asked about their one most painful region, neck, shoulder and low-back pain were the most common complaints, and only two patients who had been exposed to falanga reported their pain as being most severe in the feet. Data of reported pain and sensory symptoms for all individuals are presented separately in Tables 1, 2, 3 and 4. We have chosen to present individual data from the left feet in this paper, since we have previously demonstrated that there is no marked difference between the two sides [10]. 

**Table 1:** Reported pain, sensory symptoms, pain at rest, pain when walking and type of foot pain sorted by ‘pain when walking’ in the left foot sole of 32 torture victims (blank space=normal)

**Patient code**	**Falanga and**	**Numbness**	**Cold sensation**	**Burning sensation**	**Pricking or buzzing sensation**	**Dysaesthesia**	**Allodynia**	**Pain at rest**	**Pain when walking**	**Type of foot pain**
	**No Falanga**									
301	Falanga	yes	yes	.	yes	yes	.	slight/moderate	severe	evoked pain
302	Falanga	.	yes	yes	yes	yes	yes	slight/moderate	severe	evoked pain
303	Falanga	.	.	yes	.	yes	.	no	severe	evoked pain
304	No Falanga	yes	yes	yes	.	.	.	slight/moderate	severe	evoked pain
306	No Falanga	yes	.	yes	.	yes	.	slight/moderate	severe	evoked pain
307	No Falanga	yes	yes	yes	yes	.	.	slight/moderate	severe	evoked pain
308	No Falanga	yes	yes	yes	yes	.	.	no	severe	evoked pain
309	Falanga	.	.	yes	yes	.	.	slight/moderate	severe	evoked pain
311	Falanga	.	yes	yes	.	.	.	no	severe	evoked pain
312	Falanga	.	.	.	.	.	.	no	severe	evoked pain
313	No Falanga	.	.	.	yes	yes	yes	slight/moderate	severe	evoked pain
314	No Falanga	yes	yes	yes	yes	yes	.	slight/moderate	severe	evoked pain
316	Falanga	yes	.	yes	yes	yes	.	slight/moderate	severe	evoked pain
317	No Falanga	yes	yes	yes	yes	yes	.	severe	severe	independent pain
322	Falanga	.	.	yes	yes	.	.	slight/moderate	severe	evoked pain
324	Falanga	yes	.	.	.	yes	.	slight/moderate	severe	evoked pain
326	No Falanga	yes	yes	yes	yes	yes	.	slight/moderate	severe	evoked pain
327	Falanga	yes	.	yes	yes	yes	.	slight/moderate	severe	evoked pain
328	Falanga	yes	yes	yes	yes	.	.	slight/moderate	severe	evoked pain
332	No Falanga	.	yes	.	yes	.	.	slight/moderate	severe	evoked pain
333	No Falanga	yes	.	yes	yes	yes	.	severe	severe	independent pain
337	Falanga	yes	yes	yes	yes	yes	.	slight/moderate	severe	evoked pain
338	Falanga	yes	.	yes	yes	.	.	slight/moderate	severe	evoked pain
339	Falanga	yes	yes	yes	yes	.	.	severe	severe	independent pain
310	No Falanga	yes	.	.	.	yes	yes	no	slight/moderate	evoked pain
319	Falanga	.	.	.	.	.	.	slight/moderate	slight/moderate	independent pain
330	No Falanga	yes	.	yes	yes	yes	.	no	slight/moderate	evoked pain
315	No Falanga	yes	yes	yes	.	.	.	no	no	no pain
323	No Falanga	.	yes	.	.	.	.	no	no	no pain
325	No Falanga	.	.	.	.	.	.	no	no	no pain
334	No Falanga	.	.	.	.	.	.	no	no	no pain
335	No Falanga	.	.	.	.	.	.	no	no	no pain

**Table 2:** **Categorized sensory functions from QST data (hypo, normal=blank space, HYPER; see text), current pain intensity and pain area sorted by ‘pain when walking’ in the left foot sole of 32 torture victims (see Table**1)

**Patient code**	**MDT**	**CDT**	**WDT**	**CPT**	**HPT**	**PPT**	**Wind-up 0.3 Hz**	**Wind-up 1.0 Hz**	**VAS**	**Pain area**
301	Hypo	.	.	.	.	HYPER	.	.	.	56
302	Hypo	.	.	Hypo	.	HYPER	HYPER	HYPER	95	52
303	Hypo	.	.	.	.	HYPER	.	.	0	46
304	Hypo	Hypo	Hypo	Hypo	.	.	Hypo	.	52	58
306	Hypo	Hypo	.	.	.	HYPER	.	.	15	22
307	Hypo	Missing	Missing	Missing	Missing	HYPER	.	.	33	33
308	Hypo	Hypo	Hypo	Hypo	.	HYPER	Hypo	.	29	1
309	Hypo	.	Hypo	.	.	HYPER	.	Missing	34	53
311	.	.	.	.	.	.	.	.	0	0
312	Hypo	.	.	.	.	.	.	.	0	6
313	Hypo	Hypo	.	.	.	HYPER	.	.	30	19
314	Hypo	Hypo	Hypo	Hypo	.	.	.	.	46	14
316	.	.	.	.	.	.	.	.	50	36
317	Hypo	.	Hypo	.	.	HYPER	.	.	47	34
322	.	.	.	HYPER	.	HYPER	Missing	Missing	53	47
324	Hypo	Hypo	Hypo	Hypo	.	.	Hypo	.	89	89
326	.	.	.	.	.	HYPER	.	.	100	3
327	.	.	.	HYPER	HYPER	HYPER	.	HYPER	65	70
328	Hypo	.	Hypo	.	.	HYPER	.	.	47	74
332	.	Hypo	.	Missing	Missing	HYPER	Missing	Missing	47	65
333	Hypo	Hypo	Hypo	Hypo	.	Hypo	Hypo	.	37	47
337	Hypo	Hypo	Hypo	.	.	.	Hypo	.	59	40
338	Hypo	Hypo	Hypo	.	.	.	Hypo	.	10	88
339	Hypo	Hypo	.	.	.	.	Hypo	.	82	19
310	.	Hypo	Hypo	Hypo	.	HYPER	.	.	0	35
319	.	Hypo	HYPER	.	HYPER	HYPER	Hypo	.	10	28
330	Hypo	Hypo	Hypo	.	.	HYPER	Hypo	.	20	36
315	Hypo	Hypo	Hypo	Hypo	.	HYPER	.	.	0	0
323	.	Hypo	Hypo	.	.	.	.	.	0	0
325	.	.	.	.	.	.	.	.	0	0
334	Hypo	.	.	HYPER	.	HYPER	Hypo	.	0	0
335	Hypo	.	.	.	.	HYPER	Hypo	.	0	0

**Table 3:** **Reported pain, sensory symptoms, pain at rest, pain when walking and type of foot pain sorted by ‘PPT, pain when walking and type of foot pain’ in the left foot sole of 32 torture victims (blank space=normal) (see Table**4)

**Patient code**	**Falanga and**	**Numbness**	**Cold sensation**	**Burning sensation**	**Pricking or buzzing sensation**	**Dysaesthesia**	**Allodynia**	**Pain at rest**	**Pain when walking**	**Type**
	**No Falanga**									
306	No Falanga	yes	.	yes	.	yes	.	slight/moderate	severe	evoked pain
307	No Falanga	yes	yes	yes	yes	.	.	slight/moderate	severe	evoked pain
308	No Falanga	yes	yes	yes	yes	.	.	no	severe	evoked pain
313	No Falanga	.	.	.	yes	yes	yes	slight/moderate	severe	evoked pain
326	No Falanga	yes	yes	yes	yes	yes	.	slight/moderate	severe	evoked pain
332	No Falanga	.	yes	.	yes	.	.	slight/moderate	severe	evoked pain
301	Falanga	yes	yes	.	yes	yes	.	slight/moderate	severe	evoked pain
302	Falanga	.	yes	yes	yes	yes	yes	slight/moderate	severe	evoked pain
303	Falanga	.	.	yes	.	yes	.	no	severe	evoked pain
309	Falanga	.	.	yes	yes	.	.	slight/moderate	severe	evoked pain
322	Falanga	.	.	yes	yes	.	.	slight/moderate	severe	evoked pain
327	Falanga	yes	.	yes	yes	yes	.	slight/moderate	severe	evoked pain
328	Falanga	yes	yes	yes	yes	.	.	slight/moderate	severe	evoked pain
317	No Falanga	yes	yes	yes	yes	yes	.	severe	severe	independent pain
310	No Falanga	yes	.	.	.	yes	yes	no	slight/moderate	evoked pain
330	No Falanga	yes	.	yes	yes	yes	.	no	slight/moderate	evoked pain
319	Falanga	.	.	.	.	.	.	slight/moderate	slight/moderate	independent pain
315	No Falanga	yes	yes	yes	.	.	.	no	no	no pain
334	No Falanga	.	.	.	.	.	.	no	no	no pain
335	No Falanga	.	.	.	.	.	.	no	no	no pain
304	No Falanga	yes	yes	yes	.	.	.	slight/moderate	severe	evoked pain
314	No Falanga	yes	yes	yes	yes	yes	.	slight/moderate	severe	evoked pain
311	Falanga	.	yes	yes	.	.	.	no	severe	evoked pain
312	Falanga	.	.	.	.	.	.	no	severe	evoked pain
316	Falanga	yes	.	yes	yes	yes	.	slight/moderate	severe	evoked pain
324	Falanga	yes	.	.	.	yes	.	slight/moderate	severe	evoked pain
337	Falanga	yes	yes	yes	yes	yes	.	slight/moderate	severe	evoked pain
338	Falanga	yes	.	yes	yes	.	.	slight/moderate	severe	evoked pain
339	Falanga	yes	yes	yes	yes	.	.	severe	severe	independent pain
323	No Falanga	.	yes	.	.	.	.	no	no	no pain
325	No Falanga	.	.	.	.	.	.	no	no	no pain
333	No Falanga	yes	.	yes	yes	yes	.	severe	severe	independent pain

**Table 4:** Categorized sensory function from QST data (hypo, normal=blank space, HYPER; see text), current pain intensity and pain area sorted by ‘PPT, pain when walking and type of foot pain’ in the left foot sole of 32 torture victims

**Patient code**	**MDT**	**CDT**	**WDT**	**CPT**	**HPT**	**PPT**	**Wind-up 0.3 Hz**	**Wind-up 1.0 Hz**	**VAS**	**Pain area**
306	Hypo	Hypo	.	.	.	HYPER	.	.	15	22
307	Hypo	Missing	Missing	Missing	Missing	HYPER	.	.	33	33
308	Hypo	Hypo	Hypo	Hypo	.	HYPER	Hypo	.	29	1
313	Hypo	Hypo	.	.	.	HYPER	.	.	30	19
326	.	.	.	.	.	HYPER	.	.	100	3
332	.	Hypo	.	Missing	Missing	HYPER	Missing	Missing	47	65
301	Hypo	.	.	.	.	HYPER	.	.	.	56
302	Hypo	.	.	Hypo	.	HYPER	HYPER	HYPER	95	52
303	Hypo	.	.	.	.	HYPER	.	.	0	46
309	Hypo	.	Hypo	.	.	HYPER	.	Missing	34	53
322	.	.	.	HYPER	.	HYPER	Missing	Missing	53	47
327	.	.	.	HYPER	HYPER	HYPER	.	HYPER	65	70
328	Hypo	.	Hypo	.	.	HYPER	.	.	47	74
317	Hypo	.	Hypo	.	.	HYPER	.	.	47	34
310	.	Hypo	Hypo	Hypo	.	HYPER	.	.	0	35
330	Hypo	Hypo	Hypo	.	.	HYPER	Hypo	.	20	36
319	.	Hypo	HYPER	.	HYPER	HYPER	Hypo	.	10	28
315	Hypo	Hypo	Hypo	Hypo	.	HYPER	.	.	0	0
334	Hypo	.	.	HYPER	.	HYPER	Hypo	.	0	0
335	Hypo	.	.	.	.	HYPER	Hypo	.	0	0
304	Hypo	Hypo	Hypo	Hypo	.	.	Hypo	.	52	58
314	Hypo	Hypo	Hypo	Hypo	.	.	.	.	46	14
311	.	.	.	.	.	.	.	.	0	0
312	Hypo	.	.	.	.	.	.	.	0	6
316	.	.	.	.	.	.	.	.	50	36
324	Hypo	Hypo	Hypo	Hypo	.	.	Hypo	.	89	89
337	Hypo	Hypo	Hypo	.	.	.	Hypo	.	59	40
338	Hypo	Hypo	Hypo	.	.	.	Hypo	.	10	88
339	Hypo	Hypo	.	.	.	.	Hypo	.	82	19
323	.	Hypo	Hypo	.	.	.	.	.	0	0
325	.	.	.	.	.	.	.	.	0	0
333	Hypo	Hypo	Hypo	Hypo	.	Hypo	Hypo	.	37	47

### Normality criterion

Since the results from measuring sensory modalities with QST have varying denominations, we have chosen to transform the data into three categories in relation to our matched controls: hypoesthesia, normoesthesia and hyperesthesia/hyperalgesia (Tables 2 and 4). The normality values were derived from our control group data [10], setting normality as the mean +/− 1.28SD, thus including 80% of all values. In fact, this criterion produced normality data (see ranges in Table 5) quite close to those in the literature. Thus, Bell-Krotoski et al. [23] and Jeng et al. [16], found the normal values for MDT of the foot sole to correspond to a mean filament size of 3.61 and 3.57 respectively, equal to a target force of 0.4 g. As regards thermal thresholds the normal values for feet are only available from the dorsum of the foot and come from the studies of Yarnitsky et al. [24], Hagander et al. [13] and Rolke et al. [14]. For CDT the mean thresholds were 29.5°C, 31.4°C and 30.3°C, respectively. Normal mean values in the literature for HPT in the feet are (45.5°C: Yarnitsky et al. [25]; 43.7°C: Hagander et al. [13]; 47.0°C: Rolke et al. [14]). With mechano-nociceptive stimulation, Messing et al. [26] found a mean PPT of 344 kPa in the arch of the foot, whereas Rolke et al. [14] found a mean PPT of 572 kPa in the dorsum of the foot. The wind-up effect elicited with 0.3 Hz stimuli was 15 mm in normal subjects (Defrin et al. [9]). 

**Table 5:** Sensory functions in the left foot of 14 healthy controls (mean, SD, 95% CI, median and range for eight QST variables) and our calculated normality criteria (see text)

	**MDT target force g**	**CDT °C**	**WDT °C`**	**CPT °C**	**HPT °C**	**PPT kPa**	**Wind-up mm at 0.3 Hz**	**Wind-up mm at 1.0 Hz**
	**Mean**	**Mean**	**Mean**	**Mean**	**Mean**	**Mean**	**Mean**	**Mean**
	**SD**	**SD**	**SD**	**SD**	**SD**	**SD**	**SD**	**SD**
	**CI**	**CI**	**CI**	**CI**	**CI**	**CI**	**CI**	**CI**
	**Median**	**Median**	**Median**	**Median**	**Median**	**Median**	**Median**	**Median**
	**(Range)**	**(Range)**	**(Range)**	**(Range)**	**(Range)**	**(Range)**	**(Range)**	**(Range)**
Controls n=14 (left foot)	0.33	28.0	40.9	21.1	47.0	456	17	27
	0.34	2.4	3.1	4.7	2.5	167	13	22
	0.14 – 0.53	26.6 – 29.3	39.1 – 42.7	18.3 – 23.8	45.5 – 48.4	359 – 552	10 – 24	14 – 39
	0.16	28.9	41.3	20.6	47.6	461	18	26
	(0.07 – 1.00)	(21.6 – 30.2)	(36.1 – 45.1)	(9.2 – 27.9)	(42.0 – 49.8)	(208 – 783)	(0 – 39)	(−2 – 63)
Normality criterion range	0 - 0.76	24.9 – 31.1	36.9 – 44.9	15.1 – 27.1	43.8 – 50.0	242 – 670	1 – 34	0 – 55

*MDT*, mechanical detection threshold; g, gram; *CDT*, cold detection threshold; *WDT*, warm detection threshold; *CPT*, cold pain threshold; *HPT*, heat pain threshold; *PPT*, pressure pain threshold; kPa, kiloPascal; Wind-up mm at 0.3 Hz (mean pre/post difference in pain intensity VAS mm after wind-up stimulation at 0.3 Hz); Wind-up mm at 1.0 Hz (mean pre/post difference in pain intensity VAS mm after wind-up stimulation at 1.0 Hz).

### Sensory profiles of pain, symptoms and QST data in the feet

Since torture victims often present with walking difficulties, we chose to sort our patients according to how intense the reported pain in their feet was when walking (severe, slight/moderate or none; Tables 1 and 2). In the following text, all patient findings are represented by the left foot.

As can be seen in Table 1, most of our patients, irrespective of exposure to falanga, reported severe pain when walking. Such severe pain was often accompanied by sensory symptoms like burning or pricking and sometimes with dysesthesia also at rest. In Table 2 it can be seen that severe pain when walking was often associated with hyperalgesia to deep mechanical pressure (PPT) but only occasionally to current high pain intensity at rest (VAS). Hypoesthesia to mechanical stimuli (MDT) co-occurred with numbness and burning and with deep mechanical hyperalgesia (PPT) more often than not, but otherwise, a hypoesthesia to cutaneous sensory modalities did not co-occur systematically to foot trauma (falanga), pain or sensory symptoms (Tables 1 and 2). Moreover, an exaggerated wind-up reaction was rare in these patients.

### Re-arrangement of data to deepen analysis

A further analysis, re-arranging the data according to: 1) the occurrence of deep hyperalgesia, 2) pain when walking and 3) type of foot pain, more clearly illustrates that this change of sensory transmission was as common after generalized torture as after falanga (Tables 3 and 4). Furthermore, out of the 20 patients with hyperalgesia to deep mechanical pressure only one patient did not have any form of cutaneous hypoesthesia. Regarding the relation to foot trauma 10/15 falanga victims and 12/17 victims of generalized torture had cutaneous mechanical hypoesthesia (MDT). Moreover, hypoesthesia to mechanical detection was associated with hypoesthesia to cold detection in 12/22 cases and with warm- and cold-detection hypoesthesia in 9/22 cases. These differences were not significant (Fisher’s test).

## Discussion

### Pain in torture victims

From the present analysis it appears that little discriminates pain mechanisms in torture victims who have been exposed to trauma from torture on different parts of the body. Rather, it seems as if there are important overriding mechanisms, manifested by the hyperalgesia to pressure pain in the QST analysis, which is usually considered a sign of centralization [27] and is also present in fibromyalgia and whiplash associated disorders [28,29]. In addition there were frequently signs of cutaneous hypoesthesia, but since there was no obvious correlation to the localization of trauma, it may be speculated that these findings also indicate centrally evoked disturbances in sensory transmission, that is, central inhibition [11]. In fact, other researchers found, in a large study of Complex Regional Pain Syndromes, that a combination of sensory loss and deep mechanical hyperalgesia was present in the majority of the patients (66 -69%; [11]). Like in their study, the large inter-individual variation of our QST findings makes it important to present data individually rather than as group means.

Regarding torture victims, our findings are on one hand similar to those of Olsen et al. [7], who found that the pain was predominantly located where the trauma had hit the body (‘a local sign’); similarly, our patients who had been exposed to falanga had more intense foot pain and larger pain areas in the feet than those exposed to generalized torture. However, comparing the two tortured groups (generalized torture vs. falanga with generalized torture) we had expected that there would be substantial local differences, also in their sensory functions as examined by QST. This was not the case. Rather, we found indications that central, more than peripheral, mechanisms seem to play a critical role in these pain conditions.

Regarding the use of QST in medico-legal matters, such practice is not applicable, since the size of the effects of non-organic factors on the QST method is currently unresolved [30]. On the other hand, QST can assess both large and small fiber function as illustrated in the individual profiles.

The high side-to-side correlations of all QST parameters predict that short-term test-retest reliability within the same day should be high. However, formal determination of test-retest reliability over different time ranges of 1 day, 1 month or 1 year are not available [14].

### Study limitations

The patients were refugees with residence permits in Denmark and had been referred by their general practitioner to our specialized clinic, making our sample highly selected, and thus it may not be representative of all torture victims.

With our traumatized patients, it was unfortunately not possible to collect exact information on the extent of torture. Attempts to retrieve such information may produce intense anxiety and flash backs. Moreover, all patients had been subjected to various forms of torture, increasing the risk of brain injuries [31], which may contribute to the pain reported. Torture victims are vulnerable, often forget and have difficulties in focusing attention [32]. Using QST requires cooperation from the patients and the dimensions of cognitive effects on QST findings are not resolved [30,33]. Indeed, there are recent indications that chronic widespread pain is associated with lower cognitive processing speed [34]. Furthermore, the need to use interpreters in a psychophysical study, especially in a population with high levels of anxiety and depression, may present certain problems [35].

Since many of these patients have been imprisoned for long time periods, it is important to distinguish the present findings from those elicited by peripheral neuropathy, whether from toxic, nutritional or infectious causes. However, the victims were carefully examined for such comorbidities during the medical assessment (see Methods) and secondly**,** motor deficits, typical for severe polyneuropathies, were never found among the included patients. Furthermore, the combination often seen in our patients, cutaneous hypoesthesia, normal nociceptive transmission and deep mechanical hyperalgesia, is not typical for peripheral neuropathies but has been reported without signs of nerve injury in, for example, chronic regional pain syndrome [11; Appendix A in 27]. However it is difficult to completely exclude the possibility of nerve injury here.

For patients with neuropathic pain, some of the QST procedures may be briefly painful, for example the Wind-up test. In our study design, we have considered this problem, and as is obvious from our methods, the patients were always in control. In fact, we only had to discontinue one of the sensory examination sessions due to pain.

## Conclusion

In conclusion, by comparing QST data from the foot soles of 32 torture victims in chronic pain to ethnically matched healthy persons, we found hyperalgesia to deep mechanical pressure in combination with cutaneous hypoesthesia to non-nociceptive modalities to be a common sensory disturbance. We interpret these findings as a sign of changes in central sensory processing which may be the unifying pathological mechanism of chronic pain in these persons.

## Competing interests

The authors declare that they have no competing interests.

## Authors’ contributions

KP has been active in all aspects of the study: conception, design, acquisition of data (main responsibility), analysis and interpretation of data, writing of the manuscript and final approval. ALP contributed to the conception, design, analysis, and interpretation of data (main responsibility), writing of manuscript and final approval. BHS had main responsibility for the conception and design of the study, contributed to the interpretation of data and participated in writing of manuscript and final approval.

## Authors’ information

KP, PT, MSc, has long experience of treating victims of torture since more than twenty years at our centre. This research is intended to become part of her PhD - thesis. ALP, PT, PhD, is a Senior Researcher in pain and rehabilitation research at our centre and has extensive experience of multidisciplinary pain rehabilitation. BHS, MD, DMSc, Professor of Rehabilitation at the University of Southern Denmark, is a pain management physician and was till recently Director General of our centre.

## Pre-publication history

The pre-publication history for this paper can be accessed here:

http://www.biomedcentral.com/1472-698X/12/40/prepub
